# Holistic Approaches to Plant Stress Alleviation: A Comprehensive Review of the Role of Organic Compounds and Beneficial Bacteria in Promoting Growth and Health

**DOI:** 10.3390/plants13050695

**Published:** 2024-02-29

**Authors:** Sandamali Harshani Kumari Hathurusinghe, Ugur Azizoglu, Jae-Ho Shin

**Affiliations:** 1 Department of Applied Biosciences, Kyungpook National University, Daegu 41566, Republic of Korea; 2 Department of Crop and Animal Production, Safiye Cikrikcioglu Vocational College, Kayseri University, Kayseri 38039, Turkey; azizoglu@kayseri.edu.tr; 3 Genome and Stem Cell Research Center, Erciyes University, Kayseri 38039, Turkey; 4 Department of Integrative Biology, Kyungpook National University, Daegu 41566, Republic of Korea; 5 NGS Core Facility, Kyungpook National University, Daegu 41566, Republic of Korea

**Keywords:** beneficial bacteria, organic compounds, stress alleviation, growth promotion, biocontrol

## Abstract

Plants select microorganisms from the surrounding bulk soil, which act as a reservoir of microbial diversity and enrich a rhizosphere microbiome that helps in growth and stress alleviation. Plants use organic compounds that are released through root exudates to shape the rhizosphere microbiome. These organic compounds are of various spectrums and technically gear the interplay between plants and the microbial world. Although plants naturally produce organic compounds that influence the microbial world, numerous efforts have been made to boost the efficiency of the microbiome through the addition of organic compounds. Despite further crucial investigations, synergistic effects from organic compounds and beneficial bacteria combinations have been reported. In this review, we examine the relationship between organic compounds and beneficial bacteria in determining plant growth and biotic and abiotic stress alleviation. We investigate the molecular mechanism and biochemical responses of bacteria to organic compounds, and we discuss the plant growth modifications and stress alleviation done with the help of beneficial bacteria. We then exhibit the synergistic effects of both components to highlight future research directions to dwell on how microbial engineering and metagenomic approaches could be utilized to enhance the use of beneficial microbes and organic compounds.

## 1. Introduction

### 1.1. A Brief Overview of the Rhizosphere Microbiome

A microbial community confined into a narrow zone and affected by plant roots is generally identified as a rhizosphere microbiome [1]. It basically consists of bacteria, fungi, and oomycetes and is responsible for plant growth and health [2]. In agriculture, the rhizosphere microbiome, which is altered by host genotypes, root exudates, and plant acclimation, is important in gaining sustainable plant growth [3]. It is crucial in plant–microbial interactions for nutrient acquisition and can alter and influence plant health and productivity [4]. The rhizosphere selects microorganisms from the surrounding bulk soil and acts as a seed bank, depleting microbial diversity [5]. This function leads the rhizosphere to form a distinct core microbiome. The core microbiome around the roots contributes to plant growth and health. Understanding the interactions between plants and microorganisms in the rhizosphere involves recognizing the complex communication and interplay between them. Recent research highlighted the importance of these interactions in shaping the rhizosphere microbiome and its impact on plant growth and health under stress conditions.

### 1.2. The Importance of Organic Compounds and Beneficial Bacteria in Stress Alleviations

Plants go through many stressful conditions in their life cycle. These stresses can either be abiotic or biotic, depending on their nature. Abiotic stress can be identified as the negative effects of non-living factors on plant growth and resilience. These factors include drought, cold, and salinity [6,7]. Biotic stress is caused by living organisms in the forms of pests, diseases caused by pathogens, and competing vegetation [8]. Both these stresses result in the reduction of crop productivity, increase the mortality rate of vulnerable plant species, reduction of species richness in ecosystems, and depletion of plant nutrient uptake [9].

To tolerate this stress, three main solutions are available at present. First, endophytic bacteria are used as a solution to biotic stress [10]. Second, management practices such as irrigation methods, soil amendments, intercropping, tillage/ sowing methods, and crop rotation are used as a solution to abiotic stress [11]. Third, understanding both biotic and abiotic stress impacts on genetic and molecular level and the production of resistant cultivars [12]. However, these solutions are either less effective or time and resource-consuming. Therefore, a combination of rhizosphere-beneficial bacteria with organic compounds and their exogenous application is suggested here as a comparatively more effective solution. The review explains the impacts of this combination in alleviating both biotic and abiotic stress.

Plants are armored with tailored microbiome in the rhizosphere, which could be beneficial. Organic compounds and beneficial bacteria play a crucial role in shaping the rhizosphere microbiome and its impact on plant growth and health.

Organic compounds are chemical compounds that consist of carbon atoms, which are covalently bonded to other atoms, such as hydrogen, oxygen, or nitrogen. These are essential components of living organisms and play a crucial role in various biological processes [13]. Organic compounds can be categorized into primary and secondary metabolites, which are further divided into volatile and non-volatile compounds [14,15]. Organic acids are directly involved in primary metabolic pathways, such as the tricarboxylic acid (TCA) cycle, and are essential for the normal growth and development of plants involved in photosynthesis, energy production, and the regulation of intracellular pH [16]. On the other hand, volatile organic compounds (VOCs) are secondary metabolites that play a major role in the emission of plant scents and are involved in a wide range of ecological functions, including defense, pollinator attraction, and stress responses [17].

These volatile organic compounds (VOCs) are secondary metabolites emitted by plants and are released from various plant tissues, including leaves, flowers, stems, and roots, and act as airborne signals by signaling between distant plant organs and between plants for its defense. Furthermore, VOCs can modify the defense system of plants for enhanced resistance to biotic and abiotic stress [18,19]. The biosynthesis of VOCs and other secondary metabolites depends on the availability of carbon, nitrogen, and sulfur in plants [16].

There are non-volatile primary metabolites, which are released by root exudates into the rhizosphere, defined as organic compounds. Root exudates are comprised of primary metabolites such as sugars, amino acids, carboxylic acids. These compounds can perform the roles of signaling molecules, attractants, stimulants, and inhibitors, thereby influencing suppression performance in the next generation of plants [20]. Organic compounds in root exudates, including organic acids, sugars, and many other metabolites, enhance nutrient supply to the soil and root microbiota, providing a readily available supply of nutrients and energy for microbial growth [21]. Root exudates such as malic acid and fumaric acid have been shown to influence the phosphorus-solubilizing ability of bacteria, and these compounds can recruit beneficial microbes to the rhizosphere, thus positively impacting plant health [22].

Bacteria that benefit plant growth and free living are referred to as plant growth-promoting rhizobacteria (PGPR) or beneficial bacteria. These bacteria promote plant growth by colonizing the plant roots and are associated with the rhizosphere, making plant–microbe interactions possible [23]. Beneficial bacteria function in three different ways in plants: synthesizing necessary compounds for the plants, facilitating the uptake of nutrients from surrounding soil, and securing the plants from diseases [24].

**Figure 1:**
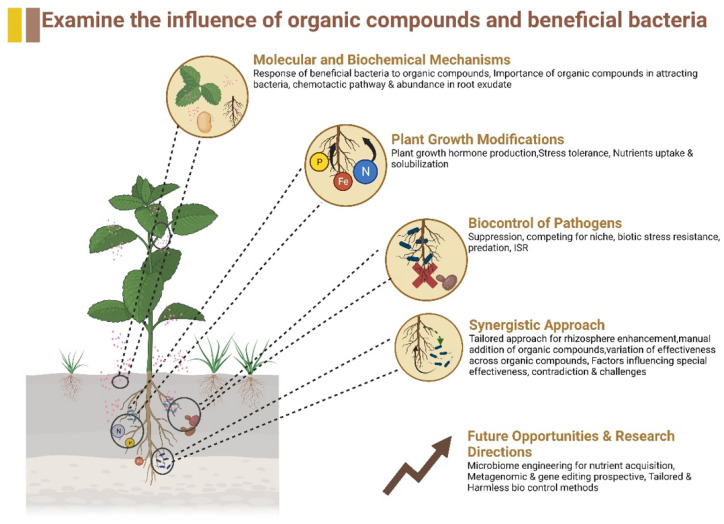
A comprehensive illustration influence of organic compounds and beneficial bacteria in enhancing plant growth and health by alleviating biotic and abiotic stress.

As this study elaborates, we need a comprehensive discussion about how these organic compounds and beneficial bacteria interplay to promote plant growth and health. Thus, this review cover five main sections on beneficial bacteria and organic compounds referring to alleviating stress, as shown in the illustration (Figure 1).

To gain a comprehensive understanding of how beneficial bacteria and organic compounds collectively mitigate biotic and abiotic stress while fostering plant growth, this study focuses on several key aspects. Initially, we delve into the molecular mechanisms and biochemical responses of beneficial bacteria to organic compounds, emphasizing regional variations and chemotactic pathways. After this, our attention shifts to the modification of plant growth facilitated by beneficial bacteria, encompassing detailed insights into growth hormone production, the enhancement of abiotic stress tolerance, and improved nutrient uptake. In this study, we further explore the regulatory role of beneficial bacteria in mitigating biotic stress through biocontrol methods. Moving forward, we investigate the synergistic effects and tailored approaches when combining both components, examining variations in the effectiveness of adding organic compounds and identifying influencing factors. Finally, we focus on future research directions, encompassing microbial engineering, metagenomic studies, gene-editing approaches, and the development of tailored and environmentally friendly biocontrol methods.

## 2. Molecular and Biochemical Mechanisms

### 2.1. The Response of Beneficial Bacteria to Organic Compounds

Depending on the environment where the beneficial bacteria live, the response shown towards organic compounds varies. 

The response of microbiota in the rhizosphere and endosphere of plants is influenced by complex molecular and biochemical mechanisms, which are characterized by the chemical nature of organic compounds and the genetic makeup of the bacteria. Plants release root exudates that promote the development of bacterial populations in the region of the rhizosphere, leading to a chemical dialogue between plants and microorganisms. This interaction by producing metabolites can improve plant response to stress conditions and promote plant function and productivity [4,25].

Primary and secondary metabolites in plants are introduced through the plant’s metabolic processes. Primary metabolites, such as glucose, are essential for the basic growth and development of plants. They also serve as precursors for the synthesis of secondary metabolites. On the other hand, secondary metabolites, including terpenoids, phenolics, and alkaloids, are not directly involved in the plant’s growth and development but play important ecological roles, such as defense and interaction with the environment [26]. First, we describe secondary metabolites, such as volatile organic compounds (VOCs). In the rhizosphere, bacterial VOCs have been found to influence plant growth and metabolic processes, potentially through their effects on gene expression and signaling pathways in plants. For example, VOCs have been found to promote plant growth and induce metabolic changes in rice [27]. In the endosphere, VOCs released by endophytic bacteria have been suggested as an alternative to chemical fertilizers and are effective in alleviating stress [28]. These VOCs belong to different chemical groups, including alcohols, ketones, benzenoids, terpenoids, sulfur-containing compounds, and alkenes. These compounds are available to bacteria by serving as energy sources, carbon, nitrogen, and other nutrient sources, as well as being toxic to the cells [29].

Root exudation is the main pathway for inputting organic carbon into soils. Here, more focus is given to primary metabolites, which are mainly organic acids, sugars, and amino acids. They affect soil physical properties, solubility, and speciation and impact the microbial community in the rhizosphere. It contains many primary and secondary plant metabolites, and the amount and composition vary depending on plant species and growth stage. As an example, mass spectrometric approaches for the analysis of root exudates were used in two different strains of *Zea mays* L., differing in root hair development. The six approaches resulted in different numbers of detected features, ranging from 176 to 889, with a fraction of 48 to 69% of significant features. All approaches showed the upregulation of most metabolites in the root hair defect [30]. Also, to find the interaction between root exudates of subterranean clover (*Trifolium subterraneum* L.) and grapevine (*Vitis vinifera* L.), an experiment was able to detect the organic acids, amino acids, and flavonoids in root exudates released by grapevine and subterranean clover grown separately and together [31].

Plant–plant interaction is another reason to produce organic compounds. A study performed on rye (*Secale cereale* L.) showed a significant increase in the production and root exudation of benzoxazinoids in response to co-cultivation with hairy vetch (*Vicia villosa*) [32].

We discussed how beneficial bacteria respond to the organic compounds in the media. Next, we are going to explore the importance of these compounds in attracting beneficial bacteria into the plant rhizosphere.

### 2.2. The Importance of Organic Compounds in Attracting Beneficial Bacteria

As shown in Table 1, different plant rhizospheres are colonized with different beneficial bacteria, while different organic compounds are present in the root exudates. The focus of this section is based on non-volatile primary metabolite organic compounds. 

A study by Sharma M et al. [33] successfully discussed a plant growth-promoting rhizobacterium such as *Bacillus velezensis* strain B26, colonized the plant roots of stiff brome (*Brachypodium distachyon*), and showed the strongest chemotactic responses in malic, succinic, citric, and fumaric acid media. In cucumber plant, two receptors that are essential components of chemotaxis, called McpA and McpC, were present with *Bacillus velezensis* SQR9 strain, while malic, fumaric, gluconic, and glyceric acids were present in media as well [34].

*Bacillus amyloliquefaciens* SQR9 and *Bacillus subtilis* N11 strains assisted in chemotaxis and biofilm formation in the roots in of cucumber and banana plants, while fumaric acid was detected in the medium [35]. Additionally, *Bacillus amyloliquefaciens* NJN-6 strain in the presence of oxalic, malic, and fumaric acids in the root exudate enhanced the root colonization of banana plants [36]. When the watermelon root exudate was saturated with naturally secreted malic, citric, succinic, and fumaric acids, it showed an enhancement in chemotaxis, swarming motility, and the biofilm formation of the same beneficial bacterium *Bacillus amyloliquefaciens* TR2 strain [37]. Also, in the tomato root exudate, malic, citric, succinic, and fumaric acids were proven to be responsible for bacterial growth, especially in the chemotactic response and biofilm formation of the *Bacillus amyloliquefaciens* T5 strain [38].

Beneficial bacterial strains of *Comamonadaceae* in cucumber enhanced their colonization in controlling fusarium wilt disease, while a machine-learning approach combined with FishTaco plus metabolic pathway analysis revealed that four organic acids (citric acid, pyruvate acid, succinic acid, and fumarate) were released at higher abundance by the Foc-susceptible cultivar compared to the resistant cultivar. Additional research revealed that *Comamonadaceae* can be cultured by these organic compounds [39].

In a multispecies consortium composed of *Bacillus* spp. strains 28 and 144, and *Microbacterium* sp. strain 50, in the stiff brome (*Brachypodium distachyon)* plant, biofilm formation was enhanced by the presence of citric acid, demonstrating how organic compounds in exudate are specifically selective in recruiting the microbiome, which is beneficial to the plant [40]. 

In addition to the above-mentioned studies, while using three organic compounds, namely L-malic, tartaric, and fumaric acids, an L-malic acid-mediated bacterial strain called *Hansschlegelia zhihuaiae* S113 showed distinct mechanisms in degrading the herbicide bensulfuron-methyl in rhizosphere soil [41]. *Hansschlegelia zhihuaiae* S113 was also capable of bioremediating the herbicide chlorimuron-ethyl in cucumber plants in the presence of citric and fumaric acids [42].

Not only did bacteria enhance their performance in the presence of organic compounds, but also biochar, a type of charcoal produced under lower oxygen levels from plant residues or animal manure, regulates mechanisms of soil improvement, nutrient management, and pH in plants, and reduces the disease incidence of bacterial wilt caused by pathogen *Ralstonia solanacearum* by 61–78% in tomato plants in the presence of citric acid and lysine (amino acid) [43].

As we study the responses of these beneficial microbes to organic compounds, a logical progression leads us to explore the chemotactic pathways and abundance in root exudates. The root exudate, rich in an array of compounds, serves as a key influencer in the behavior of beneficial bacteria. By investigating this, we aim to provide a comprehensive understanding of how plants strategically enhance biochemical cues in their root exudates to attract and modulate the behavior of beneficial bacteria.

### 2.3. Chemotactic Pathways and Abundance in Root Exudates

Chemotactic pathways and abundance in root exudates for organic compounds are important for the following reasons. These pathways of organic compounds and their abundance in root exudate facilitate the colonization of plant roots by beneficial bacteria, which can enhance plant growth, disease suppression, and stress tolerance [44]. These organic compounds can contribute to the overall structure of the microbial community in the rhizosphere, which influences nutrient mobility, microbial population, and microbial diversity [45]. These chemotactic pathways and abundance in root exudates for organic compounds can be described in detail as shown below.

The chemotaxis of beneficial rhizobacteria in root exudates contains primary and secondary metabolites, including amino acids, organic acids, sugars, alcohols, polyamines, and fatty acids. Chemotaxis can be described as the movements of bacteria in response to chemical gradients. In rhizobacteria, it is essential for efficient root colonization and the beneficial functions of plant growth-promoting rhizobacteria [44]. 

The identification of chemoattractants and chemorepellents is crucial, too. A chemotaxis-mediated response to root exudates, which contain 39 chemoattractants and 5 chemorepellents, including amino acids, organic acids, and sugars, was identified in root exudates of PGPR strain *Bacillus amyloliquefaciens* SQR9. A mutant strain, SQR9Δ8mcp, lacking all eight putative chemoreceptors, showed a loss of chemotactic responses to these organic compounds [46].

Concentration-dependent effects in plant root exudates can affect the chemosensory systems of bacteria. A higher concentration of some compounds in root exudates may enhance or inhibit bacterial chemotaxis. For example, maize root exudates (MRE) were shown to contain sugar acids, and their transcript levels increased for 10 chemoreceptor genes at low MRE concentrations, and the majority of receptor genes showed lower transcript levels at high MRE concentrations [45]. As mentioned, these functions involve complex interactions between organic compounds found in plant root exudates and bacterial chemoreceptors and play a crucial role in plant growth modifications and the biocontrol of pathogens. 

## 3. Plant Growth Modification

Plant growth modification involves plant hormones and growth regulators to alter various stages of plant development, such as flowering, aging, root growth, and fruit ripening. These regulators are either natural hormones produced by plants or synthetic compounds. They are used to control and modify physiological processes in plants under different stresses, including the differentiation and elongation of cells, the formation of leaves, flowers, and stems, and the ripening of fruit [47]. Furthermore, we discuss the potential of beneficial bacteria demonstrated in plant growth promotion.

### Potential of Beneficial Bacteria in Plant Growth Promotion 

Beneficial bacteria have been shown to enhance plant growth through various mechanisms [48]. The production of plant growth hormones is one of the main mechanisms. Lactic acid bacteria called *Leuconostoc* sp., which is isolated from the aerial part of a pomegranate plant, have been found to promote plant growth through the production of hormones [49].

Stress tolerance is another mechanism that beneficial bacteria assist. Beneficial bacteria help plants to cope with abiotic stress, such as salt, drought, and cold stress. In tomato plants, when subjected to salt stress, 1-aminocyclopropane-1-carboxylate (ACC) deaminase activity and trehalose accumulation of *Pseudomonas* sp. UW4 has been tested and proven to enhance crop growth and productivity under high salinity stress [50]. Drought is another common abiotic stress. Bacterial isolates of phosphorus-solubilizing rhizobacteria, *Pseudomonas libanensis*, with multifunctional plant growth-promoting attributes, were isolated from different cereal crops grown in Himachal Pradesh, addressed nutrient intake and limited water supply of wheat [51]. Cold stress causes a decrease in crop production. In wheat, a study designed to alleviate cold stress by inoculating psychrophilic PGPR bacteria belonging to *Bacillus* strains CJCL2 and RJGP41 positively improved plant growth by regulating phytohormone expressions [52].

Nutrient uptake and solubilization are other potential benefits of beneficial bacteria in plant growth promotion. Plants require different kinds of mechanisms to promote their growth, as demonstrated in Table 2. The beneficial bacteria attracted by organic compounds (secreted by plant roots) assist in such mechanisms as nitrogen fixation, phosphate solubilization, iron chelation, and micronutrient uptake (zinc, copper, and manganese).

Nitrogen (N) fixation is the process of plants up taking atmospheric nitrogen and converting it into a usable form with the help of beneficial bacteria. In bottle gourd (*Lagenaria siceraria*) and okra (*Abelmoschus esculentus*), the beneficial strain *Azotobacter* (SR-4) was identified as an efficient nitrogen fixer that increases plant height, leaf length and width, fruit size, and the number of fruit and is now used as a biofertilizer [53].

Phosphorus (P) solubilization is also a nutrient-uptake method in plants where insoluble phosphorus is converted into its soluble form. In maize plants, a significant enhancement of P intake could be seen when *Bacillus amyloliquefaciens* was combined with humic acid and the arbuscular mycorrhizal fungi strain B3HAM [54]. Also, in wheat, the beneficial bacterium *Pseudomonas* sp. MS16, which produces gluconic acid, was a suitable candidate to enhance phosphorus uptake, which would increase plant biomass in 2 consecutive years of yield [55].

Iron (Fe) is needed for chlorophyll production in plants. Other mechanisms that involve iron are electron transport, enzyme activation, and nitrogen fixation. Beneficial bacteria enhance iron availability via iron chelation and root system modifications. Milkvetch (*Astragalus sinicus*) is commonly used as green manure in farming. The soil-borne beneficial bacterium *Burkholderia cerpia* JFW 16 assists in Fe acquisition even under low iron-availability conditions of the soil. This leads to the improvement of the flavin metabolic pathways of the Milkvetch plant, demonstrating an increase in biosynthesis and secretion of flavins from roots [56].

Zinc (Zn), which regulates hormones, photosynthesis, and enzyme synthesis, is an important micronutrient in growing plants. In chickpea (*Cicer arietinum* L.), both *Bacillus* sp. strain AZ17 and *Pseudomonas* sp. strain AZ5 were found to assist in Zn uptake, which ultimately increases the grain yield, regardless of growing in fertilized or non-fertilized soil [57].

Copper (Cu) is not only an essential micronutrient for plants to regulate plant growth and reproduction, but also excessive amounts of copper in the soil can inhibit plant growth by incurring oxidative stress and reducing photosynthesis. Therefore, beneficial bacteria mediation is important in balancing the nutrient uptake of Cu. In the sunflower plant (*Helianthus annuus*), *Pseudomonas* sp. strain TR15a and *Bacillus aerophilus* strain TR15c result in root Cu uptake by 47% and shoot Cu intake by 75% than that of control [58].

Manganese (Mn) is another micronutrient that helps in plant growth by activating enzymes regulating chloroplast function and in the germination of seeds and reproduction procedures. In tomato, the beneficial bacterium *Bacillus pumilus* enhanced the Mn content by more than 29.46% compared to the control tomato fruit tested. This resulted in the highest fruit yield and improved plant growth compared to the uninoculated control [59]. 

As shown above, growing with the assistance of beneficial bacteria and organic compounds would not only be helpful for plants suppressing abiotic stress but also need to be healthy while tackling biotic stress. In this scenario, other mechanisms should be considered, such as the biocontrol of plant pathogens.

## 4. Potential of Beneficial Bacteria in Biocontrol of Pathogens

### Strategies Employed by Beneficial Bacteria

Biocontrol is a term used in a few fields of study, yet in plant pathology, it refers to either microbial antagonists that assist in suppressing diseases or using host-specific pathogens to control weeds in farming. In recent years, biocontrol has become a more popular technique as it is a more environmentally friendly and economically affordable approach in the agriculture sector. In suppressing biotic stress, especially caused by plant pathogens, the mediation of beneficial bacteria and their controlling mechanisms are discussed in the following contexts as well as in Table 3.

Suppression is the ability of beneficial microbes to hinder the growth or activity of plant pathogens. In cucumber, Fusarium wilt (CFW) is caused by the pathogen *Fusarium oxysporum* f. sp. *cucumerinum* (Foc). The beneficial bacterium *Enterobacteriaceae* suppressed its pathogen effect by 51% compared to the control. In addition, a combined mixture of beneficial bacteria *Pantoea dispersa* E318, *Pseudomonas koreensis* Ps213, and *Cronobacter* spp. C1 + C7 decreased the disease index by 77.2% and 60.0% in the pot experiment [60].

Niche competition refers to beneficial microbes competing with plant pathogens for specific ecological niches or resources in the plant environment. In winter wheat, the yellow mosaic disease caused by soil-borne Chinese wheat mosaic virus was biocontrolled by the beneficial bacteria *Streptomyces*, *Stenotrophomonas*, *Bradyrhizobium*, *Sphingomonas,* and *Bacillus*, which may have occurred by lessening the impact of the disease on plants by competing with pathogens for niches in the rhizosphere and root endosphere [61].

Biotic stress resistance in plants can be described as the ability of plants to withstand and defend against attacks by living organisms, such as pathogens. In strawberry, several cultivars that were exposed to diseases like verticillium wilt (caused by *Verticillium dahliae*) and charcoal rot (caused by *Macrophomina phaseolina*) showed high resistance against biotic stress induced by pathogens. In these highly resistant cultivars, *Actinobacteria* (*Arthrobacter, Nocardioides,* and *Gaiella*) and unclassified *Acidobacteria* (Gp6, Gp16, and Gp4) were in high abundance. Also, the pathogen *V. dahlia*-resistant cultivar rhizosphere contained the beneficial bacterium *Burkholderia,* and the pathogen *M. phaseolina*-resistant cultivar rhizosphere had the beneficial bacterium *Pseudomonas* in a higher relative abundance [62].

Predation in the context of plant–microbe interactions refers to the ability of certain microbes, such as predatory bacteria, protists, or fungi, to feed on plant pathogens. Broad beans (*Vicia faba* seedlings), which exhibit the disease fusarium rot caused by the pathogen *Fusarium solani* S55, showed that the disease could be suppressed successfully by the beneficial bacteria in combination with protists through predation. When protists (*Rosculus terrestris* S14D1, *Bodomorpha* sp. C10D3, and *Cercomonas lenta* C5D5) and beneficial bacteria (*Rahnella aquatilis* B16C and *Pseudomonas yamanorum* B12) are co-inoculated, plants are more resistant to the fungus *Fusarium solani* S55. The bacterivorous soil protists inoculated into *Vicia faba* seedlings in combination provided total defense against fusarium rot [63].

Induced Systemic Resistance (ISR) is a mechanism by which beneficial microbes induce resistance in plants against a broad spectrum of pathogens. It activates the plant’s own defense mechanisms to enhance its ability to resist subsequent pathogen attacks. Barley seed, which undergoes fungal pathogenic attacks, especially by *Blumeria graminis*, could be protected through induced systemic resistance by the endophytic beneficial bacteria *Paenibacillus*, *Pantoea,* and *Pseudomonas* spp., which resided within the seeds that later colonized the rhizosphere [64].

We have completed the discussion on the effect of beneficial bacteria mediated with organic compounds in plant growth modification and biocontrol of plant pathogens. Next, we investigate the possibility of the tailored introduction of organic compounds into plants. The aim is to uncover strong proof of enhanced growth and health of treated plants compared to non-treated ones.

## 5. Synergistic Effects of Organic Compounds and Beneficial Bacteria

In this context, rather than a single introduction of either beneficial bacteria or organic compounds, the discussion elaborates on the co-inoculation of both components. To harness a better outcome, we first focus on a tailored approach.

### 5.1. Tailored Approaches for Rhizosphere Enhancement

The synergistic effects of organic compounds and beneficial bacteria in the rhizosphere have been discussed here, highlighting the potential for a tailored approach to enhance plant growth and health. The following studies are worth mentioning to find out the effects of microbes on plant growth and health.

Research has shown that even the co-culture of rhizosphere microorganisms with other microorganisms can lead to enhanced antimicrobial activity and plant growth-promoting functions, which could be more advanced than monoculture applications. In a study, two potentially beneficial bacteria, *Streptomyces albireticuli* MDJK11 and *Streptomyces alboflavus* MDJK44, were selected to explore the effects of co-culture and monoculture on wheat plant growth promotion and disease prevention, and as compared to the monoculture, the co-culture showed the advantage of a synergistic enhancement effect by enhancing the function of dissolving phosphorus and nitrogen and promoting the growth of wheat roots and seedlings. It also showcases a better control ability over wheat, corn, and tobacco diseases infected by pathogenic fungi [65].

Fewer studies have been carried out in recent years to determine the effects of organic compounds on plant growth and health. Identifying and characterizing Zn-solubilizing strains from chickpea rhizosphere for growth promotion and Zn biofortification has resulted in revealing the production of hexanoic acid, pentanoic acid, and mandelic acids as key organic acids [66]. In another study, the production of indole-3-acetic acid by *Trichoderma* sp. RW309 contributes to plant growth during the early stage, and it also stimulates nitrogen mineralization and enhances soil phosphatase activity [67].

To discuss the synergistic effects of beneficial bacteria and organic compounds and their tailored approaches, knowing about exogenous co-inoculation is important. Exogenous co-inoculation can be referred to as an intentional introduction of beneficial bacteria and organic compounds into the parts of the plant or to the rhizosphere. This method aims to establish a favorable environment around plant roots, harnessing the synergistic effects of microbial activities and organic inputs to enhance overall plant health and growth. One way to do this is by manually adding organic compounds to attract beneficial bacteria.

### 5.2. Manual Addition of Organic Compounds to Attract Beneficial Bacteria

Some studies have been conducted on the manual introduction of organic compounds. The mentioned content applications of sugars, organic acids, plant hormones, and amino acids are shown in Table 4.

Carbohydrates, specifically sugar-based organic compounds, are commonly used in manual applications of agro-technology. In one study, a signaling cascade was triggered by an external sucrose spray, which encouraged *B. subtilis* rhizosphere colonization. The addition of sucrose to the media increased resistance to the airborne disease caused by *Botrytis cinerea* in tomatoes (gray mold) by about 80% and increased suppression efficiency against the soil-borne disease caused by *Fusarium oxysporum* sp. *Lycopersici* (Fusarium wilt) by about 51% [68]. In another study, glucose and sucrose co-inoculated the Italian ryegrass (*Lolium multiflorum*) and alfalfa (*Medicago sativa*) used as silage in animal husbandry, which changed the microorganism alpha diversity, and a significant LEfSe difference, which ultimately enhanced the silage quality [69].

Naturally found in plants, organic acids are compounds featuring one or more carboxylic functional groups. They play a crucial role in regulating plant metabolism and enhancing growth. Therefore, the exogenous addition of organic acids showcased distinct results in plant growth promotion and biocontrol under stress conditions. Malic acid and the beneficial bacterium *Bacillus amyloliquefaciens* strain ALB629, which were coated on common bean seeds (*Phaseolus vulgaris*), induced a higher growth promotion rate and higher drought tolerance in the germinated plant [70]. The application of citric acid (CA) and *Staphylococcus aureus* bacteria together in castor bean (*Ricinus communis* L.) foliar resulted in the phytoremediation of chromium (Cr) by increasing the amount of Cr3+ and Cr6+ uptake under chromium stress. The application of CA 5 mM with *S. aureus* increased the superoxide dismutase (SOD), peroxidase (POD), catalase (CAT), and ascorbate peroxidase (APX) in leaves by 35% and 49%, 43%, and 30%, 37% and 46%. Also, in roots, 65% and 46%, 48% and 43%, and 63% and 46% of increase could be observed in the presence of Cr 10 and 20 mg kg^−1^, respectively, as compared to their control [71]. 

Plant hormones considered to be organic compounds, also known as phytohormones, are chemical compounds that regulate plant growth, development, and responses to environmental stimuli. Salicylic acid (a plant hormone) and cell extracts of *Corynebacterium glutamicum* and *Saccharomyces cerevisiae* were administered on the foliage of 10-day-old rice plants. Thereafter, the colonization capacity of the PGPR strain *Pseudomonas chlororaphis* (ZSB15-M2) was detected. The ZSB15-M2 population was 100 times more abundant in the rice rhizosphere when the combination was added, and this resulted in distinguishing increased releases of total carbon, total protein, total sugar, total amino nitrogen, total nitrogen, and phenol content in root exudate [72]. Exogenous phytohormone indole acetic acid, combined with *Pseudomonas aeruginosa* and *Bacillus megaterium*, has a better impact on morphological and physiological traits in pigeon pea (*Cajanus cajan* L.) plants, which promotes osmolyte synthesis, increasing photosynthetic activity and mineral uptake in dealing with water stress [73].

Amino acids play essential roles in plant growth, development, and overall metabolism of plants. The manual application of amino acids promotes plant development. Gamma-aminobutyric acid (GABA) is a non-protein amino acid and a signaling molecule. The co-application of GABA and *Bacillus pumilus* JIZ13 significantly enhanced photosynthetic efficiency, chlorophyll accumulation, anti-oxidant levels, levels of osmotic adjustment substances, and biomass of rice [74].

Even though the above-mentioned instances discuss how the combination of organic compounds and beneficial bacteria enhance plant growth and health, mostly under stress, there are occasions where effectiveness varies. In the next subtopic, we are going to talk about the variation of effectiveness across organic compounds.

### 5.3. Variation in Effectiveness across Organic Compounds

The variance of effectiveness commonly refers to the variability of the outcome observed in a mechanism. Regarding this review, it can be described as a method of using organic compounds in collaboration with beneficial bacteria. The effectiveness of organic compounds in promoting synergistic effects with beneficial bacteria is not uniform through plants and different bacteria. Sometimes, organic compounds do not assist in bacterial attraction. A study has indicated that organic acids and transition metals can synergistically inhibit the growth of a broad range of bacteria, including pathogenic bacteria [75].

The type of compound is one of the reasons for the variance in effectiveness. Some organic compounds help some bacteria to thrive. Organic compounds like citric, oxalic, and lactic acids have been found to influence the soil bacterial community. This study reveals that oxalic acid is the major reason for the dominance of the *Burkholderiales* order and the *Oxalobacteraceae* family, as these bacteria are capable of utilizing oxalic acid [76].

Additionally, the size of the compound also matters. Small-molecule organic compounds such as citric, fumaric, succinic, and pyruvic acids were reported to impact certain beneficial bacterial groups and reported to attract *Pseudomonas fluorescens* WCS365, which are important for plant growth and disease suppression [39]. 

These findings suggest that different organic compounds can selectively influence the abundance and activity of beneficial bacteria in the rhizosphere. However, the type and the size of the organic compound itself do not only affect the activities of the rhizosphere. There are other factors, too.

### 5.4. Factors Influencing Specific Effectiveness

The effectiveness of organic compounds and beneficial bacteria on plants is not uniform. There are many factors affecting the effectiveness of this combination.

The plant species matters. Even though the treating group contains the same beneficial bacteria, different plants may have different pathogens that could be targeted for biocontrol [77].

The growth conditions of the plant, such as temperature, soil quality, and moisture, can influence the population of beneficial bacteria and the production of organic acids. Optimal growth conditions can lead to a more effective synergistic effect. 

At high-temperature stress, the application of bacteria such as *Paenibacillus*, *Azospirillum*, *Rhizobium*, *Bacillus*, *Azotobacter*, *Klebsiella*, *Pseudomonas,* and *Serratia* can enhance hormonal balance, regulate nutrient uptake, and assist in plant growth [78]. Soil qualities vary depending on factors such as hazardous agrochemicals, nutrient-deficit, saline, heavy metal contaminated soils, and problematic bioavailability of the major macronutrients. Likewise, the abilities of added beneficial bacteria to suppress pathogens could also vary [79]. Moisture (rain or high air humidity) can cause hypersensitive response cell death, which is responsible for secondary immune response, resulting in increased plant susceptibility to pathogens and making it hard to biocontrol [80]. 

Bacterial strain specificity is another factor influencing specific effectiveness. The effectiveness of organic acids and beneficial bacteria may vary depending on the specific strain of bacteria being targeted. Plants can release specific root exudates to recruit beneficial bacteria upon pathogen invasion. Some strains may be more resistant to pathogens, and in a study carried out to identify strains that resist foliar pathogen infections, ten *Pseudomonas* strains were found to be more successful [81].

The concentration of the medium is important as well. The concentration of organic acids can affect their effectiveness in promoting synergistic effects with beneficial bacteria. A salicylic acid concentration of 0.5%, exogenous co-application with beneficial bacteria *Bacillus amyloliquefaciens*, had the highest effect on the biocontrolling pathogen *Penicillium digitatum* in citrus [82].

However, organic compounds may not always be beneficial to plant growth and health. There are instances where organic compounds attract pathogens.

### 5.5. Contradictions and Challenges

Contrary to these findings, a few studies cited that pathogenic virulence was enhanced in the presence of organic compounds. One study showed that fumaric acid mediated the enhanced biofilm formation of the pathogen *Ralstonia solanacearum* in tobacco roots [83]. 

Another study indicates that malic and citric acid medium induced the pathogen *Ralstonia solanacearum* population in the tomato plant. However, adding *Bacillus amyloliquefaciens* (a beneficial bacterium) to the same medium with the above-mentioned organic compounds significantly decreased the root colonization of *Ralstonia solonacearum* [84].

Next, we focus on further opportunities and research direction in employing these organic compounds and beneficial bacteria in plant growth and health.

## 6. Future Opportunities and Research Directions

### 6.1. Microbiome Engineering for Nutrient Acquisition and Disease Resistance

In detailing future research directions, our focus extends towards cutting-edge methodologies and the newest advancements that hold significant promise in improving the field of plant stress alleviation through beneficial bacteria and organic compounds.

Microbiome engineering is an emerging biotechnological strategy to improve the growth and health of plants [85]. It involves deliberate interventions aimed at shaping the plant-associated microbial communities for enhanced growth by nutrient uptake and disease resistance under stress. Understanding microbiome manipulation is important to design next-generation microbial inoculants for targeted disease suppression and plant growth [86].

Microbiome engineering, including the direct inoculation of exogenous beneficial microorganisms and re-inoculation of ex situ enriched indigenous beneficial microorganisms [87], is being exploited as a promising approach to alleviate plant stress.

Furthermore, few studies have highlighted the potential of this approach in promoting plant health and combating abiotic stressors. The use of modern strategies, such as introducing novel bio-inoculants and the application of multi-omics technologies to modify genes, is emphasized as a reliable solution to enhance plant growth [87,88,89].

On the other hand, added synthetic fertilizer may not be totally acquired by plants. Instead, half of it would remain in the soil as organic compounds or subject to volatilization, leaching, and runoff [90]. Therefore, we require alternative solutions to reach advanced efficiency in plants’ acquisition of nutrients. Microbial biofertilizers used with host plants could mineralize, solubilize, or mobilize nutrients from organic matter while modulating the microbial community through competitive nutrient acquisition [91]. As an example, soil application of microbial biofertilizer significantly increased wheat production and enhanced soil phosphorus and potassium availability [92].

Simultaneously, advancements in microbial strain development and interventions, such as nano-enabled engineering for disease resistance, show promise in modulating plant systemic acquired resistance [93]. These strategies and advancements are significant in addressing current challenges in agriculture by offering sustainable solutions to improve crop health, productivity, and resilience under various environmental conditions.

### 6.2. Metagenomic and Genome Editing Prospective for Deeper Understanding

Metagenomics studies contribute to a comprehensive understanding of plant–microbe interactions by enabling the identification of the diverse microorganisms present in the plant’s environment. This approach has been utilized in recent studies to investigate the effects of bacteria and organic acids on plants. Shotgun metagenomics, which involves sequencing all the genetic material in each environmental sample without prior target-specific amplification, was used to analyze the diversity and nutrient pathways of endophytic bacteria in maize and sunflower, respectively. These studies reveal that organic fertilizers and the sunflower rhizosphere were associated with a higher abundance and diversity of beneficial bacteria and genes related to plant growth promotion [94,95]. Genome editing offers potential applications in tailoring microbial communities to achieve the desired outcomes in plants. This can be achieved by engineering specific genetic traits in microbial strains to enhance their beneficial effects on plant growth, nutrient uptake, and disease resistance. The process is described in a study which identified specific genes involved in plant growth promotion in the endophytic bacteria of the medicinal plant *Emilia sonchifolia* (Linn.) [96], which pave the pathway for future genome editing. 

For future research directions in both metagenomic and genome editing, the recent identification of the EH CRISPR-Cas9 system is a turning point. This gene-editing system is associated with a smaller Cas9 protein (EHCas9) that targets DNA sequences flanked by 5′-NGG-3′ PAMs in both prokaryotes and eukaryotic cells. The EH CRISPR-Cas9 system provides compact and efficient tools for precise future genome editing [97].

These studies collectively highlight the capacity of metagenomic approaches and genome editing in harnessing the beneficial effects of bacteria and organic acids on plants.

### 6.3. Tailored and Harmless Biocontrol Methods

Tailored and harmless biocontrol methods are advantageous as they can be a more precise tool for pest control. However, they also have limitations, such as being a slow process and vulnerability to environmental conditions. Especially in the rhizosphere, beneficial bacteria can act as a biocontrol agent to suppress plant pathogens. It secretes organic compounds such as antibiotics, antifungal metabolites, bacteriocins, antibacterial proteins, and enzymes to suppress plant pathogens and indirectly prevent pathogen infections by competing for nutrient niches and promoting plant growth under abiotic stress [98,99].

To identify tailored bacteria and possible organic compounds, the sequencing of genomes and analysis of gene functions, the detailed analysis of gene interactions between the host plant and the microbes, the modification of microbial genomes, and the identification of new potential microbial strains are crucial in further development [100].

Therefore, it is important to carefully select PGPR strains and consider their potential non-target effects before using them as biocontrol agents. Additionally, future investigations into the use of different strains of beneficial bacteria in combinations need to be conducted to secure additive and more synergistic interacting outcomes.

## 7. Discussion

Our comprehensive exploration has unraveled the various aspects of the interplay between organic compounds and beneficial bacteria in the rhizosphere, offering valuable insights into their collective role in plant stress alleviation while facilitating growth and health. 

The molecular and biochemical responses of beneficial bacteria to diverse organic compounds indicate the complexity of these interactions, providing a basis for targeted interventions. By revealing the chemotactic pathways and abundance of these compounds in root exudates, we gained an understanding of the communication channels that shape the rhizosphere microbiome.

Our study also highlighted the multifaceted potential of beneficial bacteria in enhancing plants and biocontrolling against pathogens. Then, the synergistic effects of organic compounds and beneficial bacteria were discussed, revealing tailored approaches for rhizosphere enhancement by giving special attention to the manual addition of organic compounds and beneficial bacteria. However, our findings also emphasize the need to address effective variations and consider other influencing factors. 

As a concluding remark, we discuss future prospects. Microbiome engineering has emerged as a promising pathway, offering various tactics for manipulating the beneficial microbiome for enhanced nutrient acquisition and disease resistance.

Metagenomic studies and genome editing present exciting possibilities for deeper and advanced future avenues, while the quest for tailored and harmless biocontrol methods signifies a commitment to precision, safety, and environmental sustainability. 

We deviate from the above information to further discuss the long-term effects of using these components as biocontrol agents, economic and scalability assessments, any other possible negative effects, and regulatory and safety considerations. 

The long-term effects of using beneficial bacteria and organic acids as biocontrol agents are influenced by various factors, including the inhomogeneous distribution and short lifespan of antimicrobial compounds and the variability in plant microbiomes and external environmental conditions [101,102]. Additionally, the inconsistency in the performance of bacterial biocontrol agents is influenced by pathosystem factors, environmental conditions, and the interactions between the plant, the pathogen, and the beneficial bacteria [103]. Efforts are still required to understand how to modulate the microbiome to improve the efficacy of biocontrol, e.g., how the host microbiota could interfere with the stability of biocontrol agents (either bacteria or organic compounds) once it is applied, specific strain and prebiotic assistance in biocontrol metabolic activities, and application timeline [104]. Even though the use of natural antagonists, such as bacteria and their combination with organic compounds, as biocontrol agents have arisen as a sustainable approach, the effectiveness in natural field conditions is far from theory. Therefore, further studies on the ecological aspect of microbial interactions should mainly be included in biocontrol research and development to improve the effective use of beneficial bacteria as biocontrol agents [105].

Insights into Economic and Scalability Assessments are important, too. The farmers’ options for biocontrol would depend on economic properties as well. The cost efficiency of buying beneficial bacteria, storage, introduction to the field, its effectiveness and durability, and ecological sustainability need to be carefully considered [106]. Another hindrance is the gaps observed between the number of screened biocontrol agents and available marketed products and the lack of negative results of studies published by researchers. This affects finding information on the lack of efficacy of some biocontrol agents, their uneven field performances, and a severe lack of information on economic aspects [107]. 

The major and potential negative effects of using beneficial bacteria and organic compounds are discussed next. Harm to non-target organisms could be a possibility in the long run. Complex interactions with plants, pathogens, and beneficial bacterial strains may lead to biological invasion by increasing the risk of erosion or complete loss of balanced diversity and the efficacy of biological control approaches if used singly and statically for a longer period [106].

For all these reasons, regulatory and safety concerns should be employed while dealing with these biocontrol agents. The production of a combination of beneficial bacteria and organic compounds for consumer usage may not be an easy task without considering regulatory and safety considerations. As described by WHO guidelines, it is important to conduct biosafety, ethics, and engagement activities and check the compliance of the product beforehand [108].

## 8. Conclusions

The use of organic compounds and beneficial bacteria has been widely exploited in recent years as an emerging field of study. Research has revealed that these can intervene in soil health, nutrient cycling, plant productivity, and the biocontrol of pathogens under stressful conditions. The molecular and biochemical mechanisms underlying these interactions have been explored and have revealed varying outcomes depending on specific interactions. The long-term effects of these interventions have demonstrated their effectiveness in improving plant growth and health. Economic and scalability assessments have shown that these interventions can be cost-effective and scalable. However, potential negative effects and regulatory and safety considerations must be carefully considered. In conclusion, the manual application of beneficial bacteria and organic compounds plays a crucial role in fostering sustainable agriculture, and further research and development in this area can contribute to the possible replacement of synthetic fertilizers and bio-pesticides in the future.

## Figures and Tables

**Table 1:** The importance of organic compounds in attracting beneficial bacteria.

Bacteria	Organic Compound in Media ^a^	Mechanism	Plant	Reference
*Bacillus velezensis*	Malic, Succinic, Citric, Fumaric	Chemotactic response	*Brachypodium distachyon*	[33]
*Bacillus velezensis*	Malic, Fumaric, Gluconic, Glyceric	Chemotactic response	*Cucumus sativus* L.	[34]
*Bacillus amyloliquefaciens* *Bacillus subtilis*	Fumaric	Chemotactic response Biofilm formation	*Cucumus sativus* L., *Musa acuminata*	[35]
*Bacillus amyloliquefaciens*	Malic, Oxalic, Fumaric	Chemotactic response Biofilm formation	*Musa acuminata*	[36]
*Bacillus amyloliquefaciens*	Malic, Citric, succinic, Fumaric	Chemotactic response Swarming motility Biofilm formation	*Citrullus lanatus*	[37]
*Bacillus amyloliquefaciens*	Malic, Citric, succinic, Fumaric	Chemotactic response Biofilm formation	*Lycopersicon esculentum*	[38]
*Comamonadaceae*	Citric, Pyruvate, Succinic, Fumarate	Enhance colonization in roots	*Cucumus sativus* L.	[39]
*Microbacterium*	Citric	Biofilm formation	*Brachypodium distachyon*	[40]
*Hansschlegelia zhihuaiae*	L-malic, Tarteric, Fumaric	Degrade herbicides	*Zea mays* L. *Cucumus sativus* L.	[41,42]

^a^ Organic compound in media—the organic compounds already exist in media due to plant or microbial secretion.

**Table 2:** Importance of beneficial bacteria in growth promotion.

Mechanism	Beneficial Bacteria	Outcome	Plant	Reference
Production of hormones	*Leuconostoc* sp.	Promote plant growth	*Punica granatum* L.	[49]
Stress tolerance	*Pseudomonas* sp. *UW4*	Reduce salt stress	*Lycopersicon esculentum*	[50]
	*Pseudomonas libanensis*	Response to drought stress	*Triticum aestivum*	[51]
	*Bacillus sp. CJCL2*, *RJGP41*	Response to cold stress	*Triticum aestivum*	[52]
N fixation	*Azotobacter*	Increase plant height, leaf length, fruit size	*Lagenaria siceraria Abelmoschus esculentus*	[53]
P solubilization	*B. amyloliquefaciens* *Pseudomonas* sp.	Increase plant biomass	*Zea mays* L. *Triticum aestivum*	[54,55]
Iron chelation	*Burkholderia cerpia*	Improve flavin metabolic pathway	*Astragalus sinicus*	[56]
Zn uptake	*Bacillus* sp. *Pseudomonas* sp.	Increase in grain yield	*Cicer arietinum* L.	[57]
Cu uptake	*Bacillus aerophilus*	Improved plant growth	*Helianthus annuus*	[58]
Mn uptake	*Bacillus pumilus*	High fruit yield Improved plant growth	*Solamum lycopersicum*	[59]

**Table 3:** Importance of attracted-beneficial bacteria in biocontrol of pathogen.

Mechanism	Beneficial Bacteria	Pathogen	Plant	Reference
Suppression	*Enterobacteriaceae*	*Fusarium oxysporum f.* sp. *cucumerinum*(Foc)	*Cucumus sativus* L.	[60]
Compete for niche	*Streptomyces, Stenotrophomonas, Bradyrhizobium, Sphingomonas Bacillus*	Chinese wheat mosaic virus	*Triticum aestivum* L.	[61]
Resistance to biotic stress	*Arthrobacter*, *Nocardioides* *Gaiella* *Burkholderia* *Pseudomonas*	*Verticillium dahliae* *Macrophomina phaseolina*	*Fragaria* × *ananassa*	[62]
Predation (with bacterivorous protists)	*Rahnella aquatilis* *P. yamanorum*	*Fusarium solani*	*Vicia faba*	[63]
*ISR*	*Paenibacillus*, *Pantoea*, *Pseudomonas* spp.	*Blumeria graminis*	*Hordeum vulgare* L.	[64]

**Table 4:** Co-inoculation of organic compounds and beneficial bacteria.

Type	Organic Compound	Beneficial Bacteria	Plant	Mechanism	Reference
Sugars	Sucrose	*B. subtilis*	*Solamum lycopersicum*	IRS against *B. cinera* Suppression against *F. oxyparum*	[68]
	Glucose Sucrose	Rhizosphere microbiome	*Lolium multiflorum*	Enhance silage quality	[69]
Organic acids	Malic	*B. amyloliquefaciens*	*Phaseolus vulgaris*	Higher growth promotion and drought tolerance	[70]
	Citric	*S. aureus*	*Ricinus communis* L.	Phytoremediation of Cr	[71]
Hormones	Salicylic	*C. glutamicum* *S. cerevisiae*	*Oryza sativa*	Abundant of beneficial bacteria in roots	[72]
	IAA	*P. aeruginosa* *B. megaterium*	*Cajanus cajan* L.	Promote osmolyte synthesis, increasing photosynthetic activity and mineral uptake	[73]
Amino acids	GABA	*B. pumilus*	Oryza sativa	Increase photosynthetic efficiency, chlorophyll accumulation, and anti-oxidant levels	[74]
